# Concept Design, Development and Preliminary Physical and Chemical Characterization of Tamoxifen-Guided-Mesoporous Silica Nanoparticles

**DOI:** 10.3390/molecules26010219

**Published:** 2021-01-04

**Authors:** Candace M. Day, Martin J. Sweetman, Shane M. Hickey, Yunmei Song, Yongjun Liu, Na Zhang, Sally E. Plush, Sanjay Garg

**Affiliations:** 1 Clinical and Health Sciences, University of South Australia, North Terrace, Adelaide, SA 5000, Australia; Candace.Day@mymail.unisa.edu.au (C.M.D.); Martin.Sweetman@unisa.edu.au (M.J.S.); Shane.Hickey@unisa.edu.au (S.M.H.); May.Song@unisa.edu.au (Y.S.); 2 Future Industry Institute, University of South Australia, Mawson Lakes, SA 5095, Australia; 3 School of Pharmaceutical Science, Shandong University, Ji’nan 250012, China; Liuyongjun@sdu.edu.cn (Y.L.); Zhangnancy9@sdu.edu.cn (N.Z.)

**Keywords:** breast cancer, tamoxifen, targeting vector, poly-ʟ-histidine, mesoporous silica nanoparticles, functionalized nanostructures, surface reactivity

## Abstract

Conventional chemotherapies used for breast cancer (BC) treatment are non-selective, attacking both healthy and cancerous cells. Therefore, new technologies that enhance drug efficacy and ameliorate the off-target toxic effects exhibited by currently used anticancer drugs are urgently needed. Here we report the design and synthesis of novel mesoporous silica nanoparticles (MSNs) equipped with the hormonal drug tamoxifen (TAM) to facilitate guidance towards estrogen receptors (ERs) which are upregulated in breast tumours. TAM is linked to the MSNs using a poly-ʟ-histidine (PLH) polymer as a pH-sensitive gatekeeper, to ensure efficient delivery of encapsulated materials within the pores. XRD, HR-TEM, DLS, SEM, FT-IR and BET techniques were used to confirm the successful fabrication of MSNs. The MSNs have a high surface area (>1000 m^2^/g); and a mean particle size of 150 nm, which is an appropriate size to allow the penetration of premature blood vessels surrounding breast tumours. Successful surface functionalization was supported by FT-IR, XPS and TGA techniques, with a grafting ratio of approximately 29%. The outcomes of this preliminary work could be used as practical building blocks towards future formulations.

## 1. Introduction

Breast cancer (BC) is one of the most fatal diseases among females worldwide, with approximately 350,000 new cases diagnosed in Europe, and 130,000 deaths reported annually [1]. In 2020, BC still remains the second leading cause of death from cancer in Australian women, with approximately 3000 deaths estimated [2]. Chemotherapeutic strategies to kill cancer cells by interfering with DNA and RNA synthesis are still the standard treatment options for solid tumours [3]. However, chemotherapies are limited by their non-specific mechanisms of action, whereby healthy cells are also affected during treatment [4]. Unlike invasive and non-selective chemotherapy, targeted therapy has emerged as a promising new treatment approach due to its ability to transport small therapeutic molecules and genetic cargoes to specific molecular targets located on tumour membranes, resulting in fewer unwanted toxic effects to healthy cells [5,6]. 

Various nanomaterials have been fabricated to carry anticancer drugs and deliver them specifically to tumours through the enhanced permeability and retention (EPR) effect, with higher accuracy than conventional chemotherapy, and fewer toxic side effects [7,8,9]. One of the most cutting-edge materials that has attracted substantial attention in the field of drug delivery are mesoporous silica nanoparticles (MSNs) [10,11,12]. These materials have pore diameters ranging from 2 to 50 nm, very high porosity, good uniformity and large pore volumes, allowing them to host large quantities of both hydrophilic and lipophilic cargo, intended for therapeutic and diagnostic applications, whilst simultaneously protecting the cargo from systemic degradation and clearance [10,11,12,13]. Small-sized MCM-41-typed MSNs with hexagonal porous channels are the most commonly employed carriers for pharmaceutical applications, due to their small size, high stability, ease of synthesis and their ability to adsorb onto the surface of red blood cells without disturbing cell membrane or morphology [14,15]. By incorporating active therapeutics within the mesopores of MSNs, a smaller dose of drugs can be administered, but is selectively delivered to breast tumours via the EPR effect over a longer period of time, with reduced off-target toxicity [16]. Additionally, active targeted drug delivery can be achieved by functionalizing the surface of MSNs with different ligand molecules such as peptides, antibodies, aptamers and growth factors, so the drug-loaded-MSNs can recognize and selectively bind to receptors on the tumour surface [17,18,19,20].

Of all new BC cases diagnosed, 80% are in response to the overexpression of estrogen receptors (ERs); therefore, they are classified as estrogen receptor positive (ER+) BC [21,22,23]. ERs are promising targets for the active delivery of anticancer agents to solid breast tumours. The use of selective estrogen receptor modulators (SERMs) to block the effects of endogenous estrogen in breast tissues by competing with estrogen for ER binding has been established as one of the most effective strategies in BC therapy [24,25]. The treatment of BC with tamoxifen (TAM) has been the gold standard of hormonal therapy since 1973 and has been used as an affordable, life-saving tool for millions of women worldwide [26]. We recently reviewed how low concentrations of TAM can be employed as a targeting vector to direct the delivery of nanosystems containing other anticancer agents to solid tumours [26]. For example, Jain and colleagues incorporated TAM molecules into the lipid bilayers of liposomes, to guide the delivery of doxorubicin to ER+ tumours, which resulted in an improved uptake of liposomal doxorubicin by tumours, and subsequently higher rates of tumour cell death [27]. In another study, Dreaden et al. also used TAM as a targeting vector, to achieve selective and targeted delivery of plasmonic gold nanoparticles into ER+ BC cells [28]. The resulting system obtained a 2.7-fold enhanced drug potency when tested on MCF-7 cell lines. Even though the targetability of TAM on ER-upregulated BC and its safety profile has been well established [24,25,26], the research effort into utilizing its application for the delivery of other therapeutic and diagnostic agents in cancer therapy has not been properly explored. To the best of our knowledge, there has not been a study in which TAM is employed as a targeting vector to direct the delivery of MSN-based nano-drug delivery systems (DDSs).

Considerable research efforts have been made towards incorporating a controlled release capacity for drug-loaded DDSs. A variety of molecules can be used to block the MSN pores and act as gatekeepers to prevent the premature leakage of drug molecules from MSNs before they reach their site of action, achieving a release-on-demand mechanism [29,30,31,32,33]. The polypeptide poly(L-histidine) (PLH) is a pH-sensitive gatekeeper (p*K*_a_ ~6.10) and offers several advantages compared to other pH-sensitive options due to its biocompatibility and efficient degradation after use [34,35,36,37]. Under biological conditions (pH 7.4), PLH molecules condense on the surface of MSNs, blocking the MSN pores and acting as gatekeepers to prevent the off-target release of the incorporated drugs [36]. Subsequently, as over time the PLH-gated nanosystems enter into the tumours via passive internalization, the PLH molecules will become protonated by the acidic tumour environment (pH ~5), resulting in the PLH molecules extending from the surface of the MSNs and opening the pores to release the drug payload [36,37]. 

**Scheme 1:**
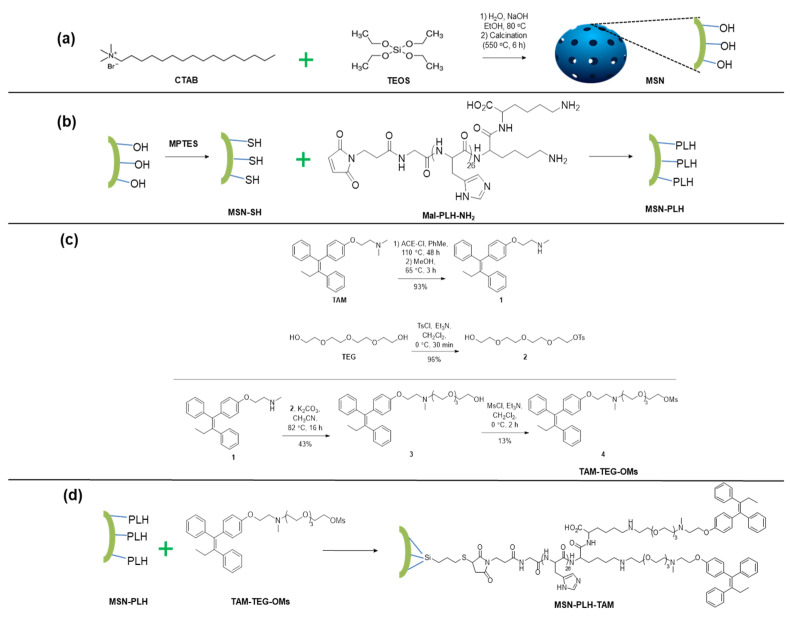
Outline of the key steps to fabricate tamoxifen (TAM)-guided- poly(L-histidine) (PLH)-gated-MSN (MSN-PLH-TAM): (**a**) Synthesis of native mesoporous silica nanoparticles (MSNs) via the Stöber method, using CTAB as the templating structure and TEOS as silica precursor. (**b**) The surface (OH-) moieties of MSNs were converted to thiols using MPTES, which were then conjugated to Mal-PLH-NH_2_ molecules via a Michael addition at pH 5, to give PLH-gated MSN (MSN-PLH). (**c**) Synthesis of TAM-TEG-OMs. (**d**) Conjugation between MSN-PLH and TAM-TEG-OMs to give the requisite MSN-PLH-TAM nanomaterial.

Here we report the conceptual design, synthesis and preliminary physical and chemical characterization of a novel nanostructure that could serve as a platform for future DDSs. The uniqueness of our functional nanostructure is derived from the combination of successful synthesis methodology and targeting strategies from previous studies in the field of DDSs. This demonstrates a new theoretical concept for a triple-action DDS to be generated, which includes: (i) passive targeting using nanosized MSNs as carriers (MCM-41 species); (ii) active targeting by employing TAM as a targeting vector to selectively bind to ER-overexpressing breast tumours; and (iii) pH-controlled drug release by grafting the polymer PLH onto the surface of MSNs to act as a pH-sensitive gatekeeper and prevent premature drug leakage. PLH and TAM are appended to the MSN in a stepwise manner, where the TAM is the peripheral moiety and is linked to the system via a non-toxic water-soluble spacer. This architecture prevents any potential π-π interaction between the hydrophobic TAM and PLH from occurring. Our system offers an exciting and novel platform for the potential development of new nanobased DDSs that could provide enhanced accuracy and lower adverse toxic effects from loaded therapeutics and diagnostic agents (Scheme 1).

## 2. Results and Discussion

### 2.1. Preparation of MSN System

The first step in the preparation of our desired triple-action functionalized nanostructure was the synthesis of the native MSNs (Scheme 1a). Small-sized MCM-41-typed MSNs with hexagonal pores were fabricated using the sol-gel approach, with surfactant cetyltrimethylammonium bromide (CTAB) used as a templating structure and tetraethoxysilane (TEOS) as a silica precursor. In aqueous solution, CTAB will self-assemble into micellar structures. As the concentration of micelles in the reaction media increases, the resulting micelles will self-assemble into hexagonal architectures. Following the addition of TEOS, silica particles will simultaneously hydrolyze and condense on the templating structure surface [10,11]. It was observed during these experiments that the concentration of NaOH played a key role in the successful formation of MSNs. When low concentrations of NaOH were employed (0.2 M to 1 M), MSNs did not form following the addition of TEOS. As the concentration of NaOH was increased to 2M, MSNs were successfully formed in the solution following TEOS addition. This phenomenon can be explained by the efficient polymerization of alkoxide monomers during the hydrolysis and condensation steps following TEOS addition. The initial hydrolysis of TEOS esters generally takes place via an S_N_2 reaction between alkoxide groups and water molecules [38]. Following the micellar formation of templating CTAB molecules, it is possible that the reactive (OH−) ions replace some of the (Br−) ions from the CTAB and attach themselves onto the micellar surface [38]. This results in increased local concentrations of (OH−) ions around the CTAB molecules in the reaction, and micelles in the Stern region [38]. Eventually, this leads to a faster and more efficient hydrolysis, which dictates the successful formation of the silica network. In the final step, removal of the surfactant template was carried out by thermal treatment (calcination). A temperature of around 550 °C must be maintained for 6 h; sufficient to remove the templating molecules but not harsh enough to disrupt the mesoporous structure.

**Figure 1:**
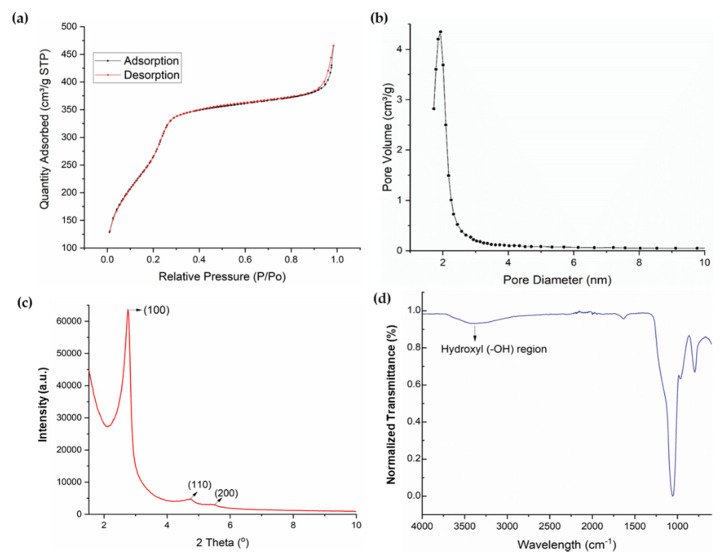
Physiochemical properties of native MSNs: (**a**) Type IV N_2_ adsorption-desorption isotherm of resulting particles, a distinct feature of mesoporous materials [39]. (**b**) Particles have an average pore diameter of 2.4 nm, which fits the definition of MSNs having pore diameters ranging from 2 to 50 nm [11,12]. (**c**) MSNs demonstrated three distinct diffraction peaks, assigned as (100), (110) and (200) planes, a characteristic crystalized lattice of MCM-41 [36,40]. (**d**) FT-IR spectra of native MSNs demonstrating the (−OH) signal [41] which is essential for further surface grafting.

The resulting MSNs displayed characteristic features of mesoporous materials, as shown by the N_2_ adsorption-desorption isotherm which demonstrated a typical type IV isotherm for mesoporous structures [39] (Figure 1a). The particles achieved a Brunauer–Emmett–Teller surface area of over 1000 m^2^/g with pore diameter at 2.4 nm and pore volume averaged at 0.79 cm^3^ g^−1^ (Figure 1b). Additionally, highly ordered mesopores and the unique crystallized lattices of MCM-41 were observed, as confirmed by the XRD measurement shown in Figure 1c. The XRD spectrum displays three distinct diffraction peaks, assigned as (100), (110) and (200) planes [36,40]. Furthermore, as demonstrated by FT-IR (Figure 1d), the MSNs display a hydroxyl (−OH) signal [41], which is crucial for further surface grafting with PLH and TAM molecules. 

**Figure 2:**
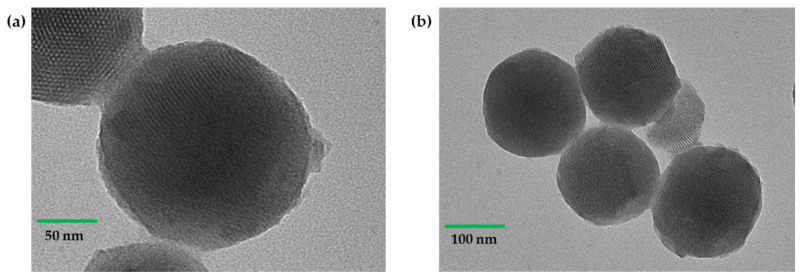
TEM images demonstrating the diameter and internal structure of MSNs: (**a**) MSNs images on the 50 nm scale-bar, demonstrating well-defined ordered long-range structure of inner mesopores. (**b**) MSNs on the 100 nm scale-bar, demonstrating highly ordered porous channels.

The fabricated MSNs were characterized to have a desirable nanosize for passive targeting, with a diameter of approximately 150 nm as determined by TEM and well-defined ordered long-range structure of inner mesopores (Figure 2a). MSNs were found to be uniform in morphology and size, with highly ordered porous channels, without the interconnection between each individual channel (Figure 2b). Dynamic light scattering (DLS) measurements suggested our MSNs were highly uniform and well dispersed; the hydrodynamic size of native MSNs was 232.97 ± 9.56 nm with an ideal PDI of 0.31 ± 0.063.

### 2.2. Synthesis of MSN-PLH

**Figure 3:**
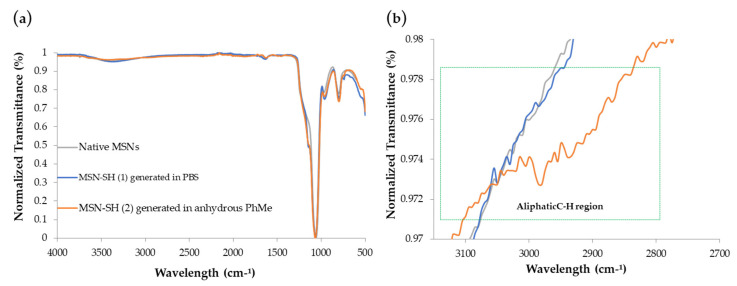
(**a**) FT-IR spectra of MSN-SH (1) synthesized in PBS pH 5 and MSN-SH (2) synthesized in anhydrous PhMe. (**b**) Enlarged region of spectrum (2800 cm^−1^ to 3150 cm^−1^) suggesting a greater presence of organic functionalities on the surface of MSN-SH (2), as compared to MSN-SH (1).

The next step in the preparation of our nanostructure was the attachment of the pH-sensitive PLH molecules to the surface of the MSNs (Scheme 1b). To achieve this, MSNs were first modified with thiol groups on the surface using 3-mercaptopropyltriethoxysilane (MPTES). Due to the sequential surface modification procedure, a higher grafting of MPTES containing SH groups on the MSNs will result in a higher coverage of PLH. Previously, the synthesis of MSN-SH has been conducted in aqueous PBS buffer (pH 5) to achieve a convenient one-spot synthesis of the subsequent MSN-PLH in the same solvent system [36]. However, there are several examples within the literature which reported similar transformation being conducted in organic solvents including toluene (PhMe) [42]. With this in mind, two solvent systems were selected to assess the grafting efficiency of SH groups onto the MSN surface using MPTES: aqueous PBS buffer (pH = 5) at 80 °C and anhydrous PhMe at 110 °C. The introduction of SH onto the native MSN systems generated in PBS buffer and in anhydrous PhMe, namely MSN-SH (1) and MSN-SH (2) respectively, were characterized using FT-IR (Figure 3a). It was observed that MSN-SH (2) showed more intense aliphatic CH_2_ peaks in the 2800 cm^−1^ to 3150 cm^−1^ region [43] (Figure 3b), indicating a higher grafting amount of the MPTES and hence higher SH coverage on the surface of MSNs when PhMe was used as the solvent. In acidic aqueous solutions there is an enhanced possibility that the alkoxysilanes (of MPTES molecules) undergo hydrolysis and self-condensation [42]. This reaction between MPTES molecules leads to polysiloxane formation [42] and, consequently, a much lower surface grafting of thiol functionalities on native MSNs. Indeed, this is observed in the MSN-SH (1) sample. Furthermore, it has been reported that the self-oxidation of attached (SH-) groups (or the reaction between thiol groups of MPTES molecules with each other) is often reduced in organic solvents such as PhMe, as compared with aqueous solvents like PBS buffer [44]. Hence, more free thiol moieties are available in anhydrous PhMe for the reaction with native MSNs. The higher reaction temperature used with the PhMe solvent may also play a significant role in the grafting ratio. As more heat-induced energy was provided, silane groups were given more opportunities to react with the (OH−) groups on the surface of native MSNs. 

To prevent surface oxidation of the newly installed SH functionalities, MSN-SH (1) and MSN-SH (2) were immediately reacted with maleimide-functionalized PLH molecules to form MSN-PLH (1) and MSN-PLH (2), respectively. Installation of the PLH group to the MSN-SH nanoparticles was achieved using a Michael addition in PBS (pH 5). It is important that PBS buffer at an acidic pH was used for the reaction of MSN-SH molecules with Mal-PLH-NH_2_. At an acidic pH, the maleimide groups are known to favour the reaction with sulfhydryl groups, resulting in stable thioether bonds [45]. In contrast, undesirable intra and inter-reactions between the maleimide groups and primary amines (from lysine groups) of Mal-PLH-NH_2_ molecules may occur at alkaline conditions, hindering the grafting of PLH onto the surface of MSNs [45].

**Figure 4:**
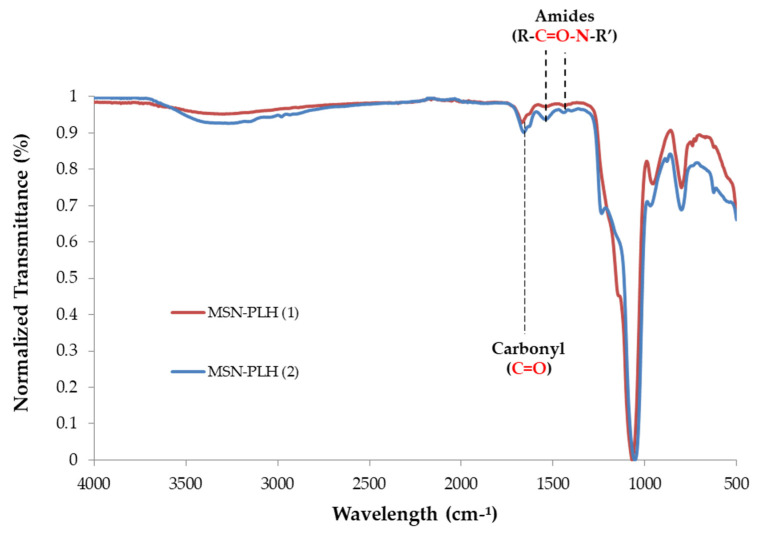
FT-IR spectra of MSN-PLH (1) and MSN-PLH (2).

The introduction of PLH onto the MSN-SH was firstly characterized using FT-IR (Figure 4). Compared to native MSNs, the FT-IR spectra of both batches of MSN-PLH compounds demonstrated new signals at 1665 cm^−1^, which is attributed to the secondary amide C=O stretching vibration, and new signals at 1535 and 1445 cm^−1^ which indicate N-H bending vibrations of the peptide bond in PLH [36], suggesting successful functionalization of both MSN-SH materials. As expected, due to the higher grafting of SH functionalities onto the MSN surface, MSN-PLH (2) appeared to have a higher grafting of PLH molecules on the surface compared to MSN-PLH (1), as indicated by the more profound peak intensities attributable to carbonyl and N-H groups.

Thermogravimetric analysis (TGA) was then employed to determine the grafting ratio of PLH molecules onto the MSNs’ surface. The data indicated a greater presence of organic molecules in the MSN-PLH (2) sample (grating ratio = 10.2%) than in the MSN-PLH (1) sample (grating ratio = 8.6%) in accordance with the FT-IR (see Materials and Method for full experimental methodology). As such, MSN-PLH (2) was selected as the lead compound for further reactions. 

### 2.3. Synthesis of TAM-TEG-OMs

Our nanostructure was developed in a modular fashion, where the targeting vector is attached to the linker as a separate entity before it is coupled to the MSN-PLH system. The aim of this design was that it would allow the targeting moiety to be altered easily in future applications. To prepare the TAM-TEG building block (Scheme 1c), the first step involved the demethylation of the commercially available TAM to form *N*-Desmethyl tamoxifen (NDMT, **1**). The synthesis of NDMT has been previously reported by Dreaden et al. [28] who heated a solution of TAM and 1-chloroethyl chloroformate (ACE-Cl) in CH_2_Cl_2_ at reflux for 48 h in near equimolar amounts before MeOH was added, and then the reaction was stirred at reflux for a further 3 h to complete the conversion. However, in our hands no product formation was observed, even when the amount of ACE-Cl was increased. When the reaction was conducted in a less volatile solvent (anhydrous PhMe), allowing the reaction temperature to be increased to 110 °C, the presence of the desirable product (NDMT) was detected using ESI-MS analysis and starting material was consumed, ultimately allowing the isolation of NDMT 1 in 93%. 

The need for a water-soluble linking group led us to the TEG linker, often used due to its commercial availability and well-established chemistry. Another reason for selecting TEG was due to the concerns regarding the potential CNS depression associated with certain ethylene glycol (EG) species, arising from their metabolites such as glycolic acids (GA), diglycolic acid (DGA) and oxalates (OX) from EG and diethylene glycol (DEG) [46]. To the best of our knowledge no evidence of TEG forming GA, DGA or OX to any significant extent has been reported. Furthermore, unlike EG and DEG, the effects of TEG on the renal system are transient and minor [46]. To label NDMT with the linker TEG, the free amine of NDMT **1** was reacted with TEG-OTs **2**, which was prepared in-house following previously reported procedures [47,48], using basic conditions in CH_3_CN at reflux to give TAM-TEG conjugation **3**. Finally, mesylation of the terminal alcohol group was achieved using methanesulfonyl chloride (MsCl) to give the requisite TAM-TEG-OMs **4** building block for attachment to the MSN material (see Materials and Method for full reaction conditions). The final mesylation step was performed in a low yield (13%), and both bromination and tosylation approaches of compound **3** were investigated; however, in each instance the desired TAM-conjugate was unable to be isolated in acceptable purity. 

### 2.4. Synthesis of MSN-PLH-TAM

**Figure 5:**
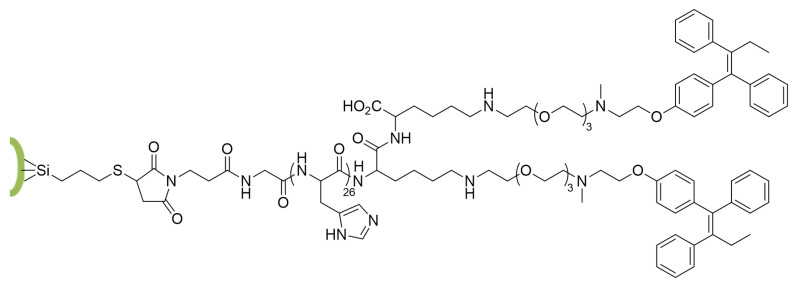
Structure of MSN-PLH-TAM.

**Figure 6:**
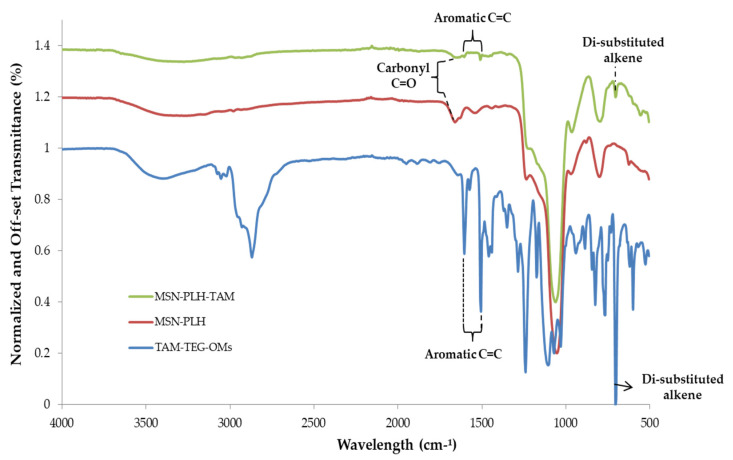
FT-IR spectra of MSN-PLH-TAM synthesized from the reaction between MSN-PLH (2) and TAM-TEG-OMs, via an S_N_2 mechanism with mesylates as leaving groups. MSN-PLH-TAM demonstrated characteristic peaks of PLH and TAM molecules, including secondary amide C=O, aromatic C=C and di-substituted alkenes.

The final step in the formation of our novel functionalized nanostructure (Scheme 1d) is the coupling of TAM-TEG-OMs to the MSN-PLH (2) building block, where the OMs group behaves as a suitable leaving group for substitution by the PLH amino groups, to achieve the desired fully functionalized MSN-PLH-TAM (Figure 5). The reaction was carried out in CH_3_CN where sonication was required to initially aid in solubilisation, before stirring was continued at 80 °C for 24 h (see Materials and Method for full reaction conditions). The characterization of MSN-PLH-TAM was firstly carried out by FT-IR analysis of MSN-PLH-TAM (Figure 6), which indicated successful conjugation of TAM-TEG-OMs to MSN-PLH (2). This was demonstrated by the appearance of peaks at 1605 cm^−1^ and 1505 cm^−1^, representing the C=C stretching of aromatic rings, and a signal at 705 cm^−1^ representing the C=C bending [49]. Additionally, the signals attributed to amide C=O groups at 1665 cm^−1^ were still present which confirmed that our final functionalized MSNs system still retained the previously grafted PLH molecules. The second characterization method employed to characterize MSN-PLH-TAM was TGA analysis. TGA was used to determine the grafting ratio of PLH and TEG onto MSNs, which further confirmed a ratio of 29% (increased from 10.2% as observed in MSN-PLH (2)), representing the presence of additional organic moieties (TAM-TEG conjugation) on the surface of the MSNs. Overall, these results obtained by FT-IR and TGA suggest that PLH and TAM were successfully grafted to the surfaces of the MSNs. As this final reaction is an S_N_2, the addition of a base was investigated. It was expected that the presence of Et_3_N would enhance the reaction efficiency to produce higher grafting of TAM molecules on the surface of MSN-PLH. Interestingly, the MSN-TAM-PLH synthesized in the presence of Et_3_N only achieved a grafting ratio of approximately 26.8%. The reduced grafting ratio may be as a result of competition between Et_3_N and PLH (from MSN-PLH) for the binding to TAM-TEG-OMs, reducing the final ratio of TAM onto PLH-MSNs. Therefore, the use of Et_3_N was discontinued for future synthesis of MSN-PLH-TAM.

**Figure 7:**
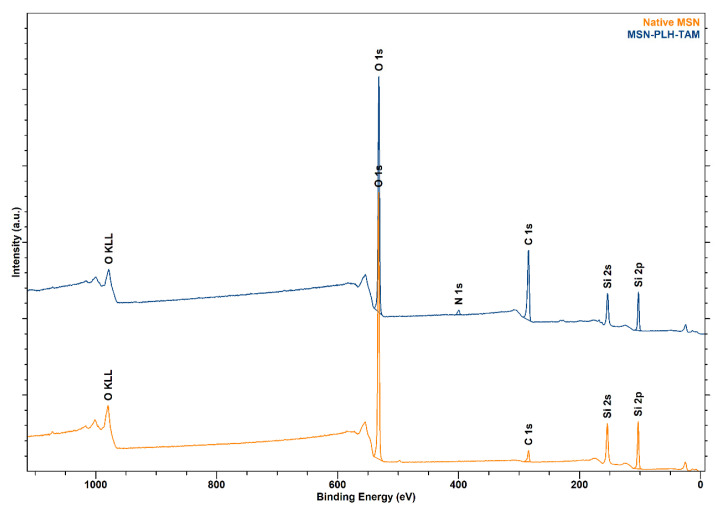
Survey XPS spectra of native MSN and MSN-PLH-TAM samples. The presence of nitrogen at ≈ 400 eV was observed in MSN-PLH-TAM sample, but not in native MSNs.

In addition to FT-IR and TGA, survey X-ray photoelectron spectroscopy (XPS) was also employed to characterize surface elements and high-resolution XPS (HR-HXS) was employed to characterize the surface functional groups of fully functionalized MSNs. When comparing the survey spectra of MSN-PLH-TAM against unfunctionalized MSNs, a signal attributable to nitrogen was observed, which was not present in the native MSNs (Figure 7). The atomic percentage of each surface element on native MSNs and MSN-PLH-TAM are reported in Table 1 below, including the absence of surface nitrogen on native MSNs and 2.2% of surface nitrogen present in the MSN-PLH-TAM sample.

**Figure 8:**
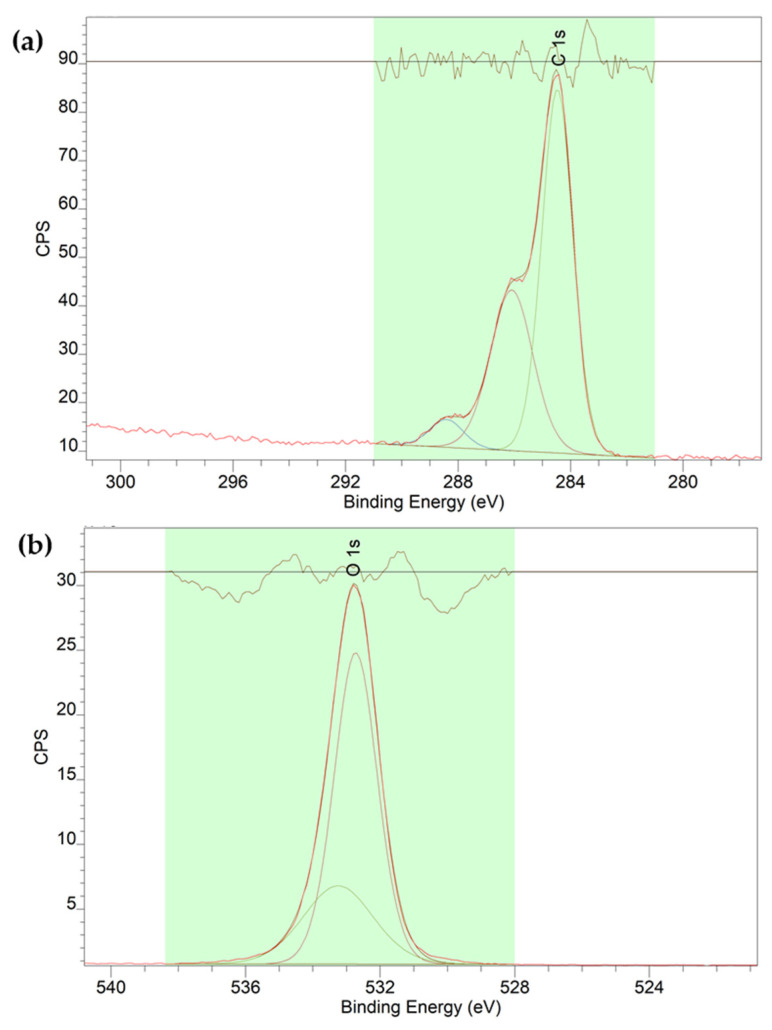
Deconvoluted HR-XPS spectra of the binding energies between carbon and oxygen are ascribed to (**a**) C 1 s and (**b**) O 1 s. Key functional groups of PLH and TAM molecules on the surface of MSNs are observed, including organic C=O and organic C-O.

In addition to survey XPS, high-resolution XPS (HR-XPS) was also employed for the determination of functional groups on the surface of obtained MSN-PLH-TAM. The spectra of carbon (C) observed on the surface of MSN-PLH-TAM was successfully fitted into three components (Figure 8a) presenting characteristic functional groups of PLH and TAM, including C=O at 288.5 eV, C-O at 286 eV and C-C at 284.8 eV [50]. HR-XPS spectra of oxygen (O) were also successfully fitted into three components (Figure 8b) representing the double bonding between carbon and oxygen, including organic C=O at 532.8 eV and organic C-O at ≈ 533 eV [51,52,53]. XPS results supported the FT-IR spectra and TGA reports for MSN-PLH-TAM, and confirmed the successful surface grafting of MSNs with both PLH and TAM.

## 3. Materials and Methods 

### 3.1. General Methods

TAM was obtained from Combi-Blocks Inc (US). PLH (Mal-PLH-NH_2_/Mal-GH26KK, purity 90%, 26 units, Mw = 4.05 kDa) was purchased from Leon Biological Technology Co. Ltd. (Nanjing, China). Anhydrous PhMe was obtained by drying over 3Å molecular sieves. All other reagents were purchased from Sigma Aldrich and used without further purification. Thin-layer chromatography was performed on silica gel 60 F_254_ plates purchased from Merch (Australia). All reactions performed at elevated temperatures were conducted using a heating mantle unless otherwise specified. All melting points were obtained using a digital ISG melting point apparatus and are uncorrected. 

All ^1^H spectra were collected on a Bruker AVANCE III 500 MHz FT-NMR spectrometer. All NMR experiments were performed at 25 °C. Samples were dissolved in either CDCl_3_ or CD_3_OD, with the residual solvent peak used as the internal reference—CDCl_3_; 7.26 (^1^H); CD_3_OD; 3.31 (^1^H) and 49.0 (^13^C) [54]. High resolution mass spectral data was collected using an AB Sciex TripleTOF 5600 mass spectrometer in a 5% H_2_O in MeOH solvent system containing 0.1% formic acid. Analyte solutions were prepared in HPLC-grade MeOH (conc. ≈ 1 mg mL^−1^).

The mesostructures and the exact diameters of native MSNs were analysed by high-resolution transmission electron microscopy (HR-TEM) (Hitachi S-4800, Tokyo, Japan) obtained on an HC electron microscope at an accelerating voltage of 100 kV. The hydrodymanic sizes of obtained particles were measured using DLS (Malvern Zetasizer Nano ZS, Worcestershire, UK). The DLS samples were prepared from a suspension of MSNs in 10^−2^ M KCl (0.1 g/L) and adjusted using a small quantity of 2 M KOH to neutral pH using a pH probe (Metrohm AG, Essen, Germany). Fourier transform infrared (FT-IR) Perkin-Elmer ATR (USA) was employed to determine the functional groups of obtained particles, with recorded spectral range from 400 to 4000 cm^−1^. To determine the crystalline structure of resulting particles, X-ray diffraction (XRD) (Malvern Panalytical Empyrean XRD, Malvern, UK) with a Cu Kα radiation source (λ = 0.15406 nm) was employed, operated at 40 kV and 30 mA. The surface area and pore volume were obtained from Brunauer–Emmett–Teller (BET) ASAP 2420 (US). Survey and HR-XPS spectrum were obtained using Kratos Analytical (Shimazdu, Kyoto, Japan), and the peak fitting was conducted using the software CasaXPS (Casa Software Ltd., Teignmouth, UK). 

### 3.2. Synthesis of Native MSNs

MCM-41 particles were synthesized following the Stöber (sol-gel) method adapted from Mu and colleagues [36]. A solution of CTAB (2.0 g, 5.49 mol) in Milli-Q water (100 mL) and 2 M NaOH (720 μL) was heated to 80 °C and stirred vigorously for approximately 30 min to allow micelles to form. TEOS (1 mL) was then added dropwise to the mixture, followed by EtOH (1 mL) and the reaction was vigorously stirred at 80 °C for a further 2 h. The resulting white precipitate was collected using centrifugation (8000 RPM, 20 min, 24 °C), and washed with H_2_O (20 mL × 3) and EtOH (20 mL × 3), before drying in a desiccator for 24 h. The resulting white particles were calcined at 550 °C for 6 h, with a heating rate of 3 °C per minute to remove the templating molecules. 

### 3.3. Synthesis of MSN-PLH

The procedure was adapted and modified from Mu et al. [36]. Native MSNs (20 mg) were dispersed in anhydrous PhMe (5 mL) and ultrasonicated for 10 min. The solution was then heated to 110 °C, and MPTES (25 µL) was added. The solution was stirred vigorously under an N_2_ atmosphere overnight. The reaction mixture was concentrated, and the white solid was washed extensively with anhydrous PhMe (20 mL) before being dissolved in PBS buffer solution (5 mL, pH 5). To the aqueous solution was added Mal-PLH-NH_2_ (20 mg) and the reaction was stirred under an N_2_ atmosphere for 24 h at room temperature. The desired solid MSN-PLH was collected from the reaction mixture using centrifugation (8000 RPM, 20 min, 24 °C), washed with PBS buffer solution (20 mL × 3) and H_2_O (20 mL × 3) and then dried in a vacuum desiccator overnight.

### 3.4. Synthesis of (Z)-2-(4-(1,2-Diphenylbut-1-en-1-yl)Phenoxy)-N-Methylethan-1-Amine (NMDT) 1 

TAM (152 mg, 0.410 mmol) was dissolved in anhydrous PhMe (6 mL) at 0 °C, followed by the addition of α-chloroethyl chloroformate (ACE-Cl) (200 µL, 1.85 mmol). After 15 min at 0 °C, the reaction was heated at reflux for 48 h under an N_2_ atmosphere. The reaction mixture was concentrated in vacuo to obtain a white waxy solid, to which MeOH (3 mL) was added and the reaction was again heated at reflux for approximately 3 h. The reaction mixture was concentrated in vacuo to give the title compound (138 mg, 93%) as a white solid. ^1^H-NMR (CD_3_OD, 500 MHz) δ 7.20–7.10 (10H, m), 6.81 (2H, d, *J* = 8.8 Hz), 6.65 (2H, d, *J* = 8.8 Hz), 4.10 (2H, t, *J* = 5.2 Hz), 3.33 (2H, t, *J* = 5.2 Hz), 2.71 (3H, s), 2.44 (2H, q, *J* = 7.5 Hz), 0.89 (3H, t, *J* = 7.6 Hz). HRMS (ESI-TOF) *m*/*z*: [M + H] calcd for C_25_H_28_NO 358.2171; found, 358.2182. Data (Figure S1 in Supplementary Materials) is in accordance with literature [28].

### 3.5. Synthesis of 2-(2-(2-(2-Hydroxyethoxy)Ethoxy)Ethoxy)Ethyl 4-Methylbenzenesulfonate 2

A solution of TEG (10.19 g, 52.4 mmol) in anhydrous CH_2_Cl_2_ (10 mL) under an N_2_ atmosphere was stirred at 0 °C for 30 min before TsCl (1.10 g, 5.24 mmol) and Et_3_N (1.1 mL, 7.89 mmol) were added sequentially. The mixture was stirred overnight before the reaction mixture was diluted in CH_2_Cl_2_ (10 mL) and transferred to a separatory funnel. The organic portion was washed with H_2_O (3 × 10 mL), dried (Na_2_SO_4_), filtered and concentrated in vacuo to give the title compound as a colourless oil (1.750 g, 96%). ^1^H NMR (CDCl_3_, 500 MHz) δ 7.78 (2H, d, *J* = 8.4 Hz), 7.33 (2H, d, *J* = 8.4 Hz), 4.14 (2H, t, *J* = 4.8 Hz), 3.59–3.70 (14H, m), 2.43 (3H, s). HRMS (ESI-TOF) *m*/*z*: [M + H] calcd for C_15_H_25_O_7_S 349.1316; found 349.1312. Data (Figure S2 in Supplementary Materials) is in accordance with literature [47].

### 3.6. Synthesis of (Z)-1-(4-(1,2-Diphenylbut-1-en-1-yl)Phenoxy)-3-Methyl-6,9,12-Trioxa-3-Azatetradecan-14-ol 3

The procedure was adapted and modified from two previously published methods [47,48]. A mixture of **1** (113. 68 mg, 0.318 mmol), **2** (132.6 mg, 0.381 mmol), K_2_CO_3_ (175.5 mg, 1.272 mmol) and CH_3_CN (4.5 mL) was stirred at reflux overnight. The reaction mixture was concentrated in vacuo to obtain a yellow oil which was diluted in CH_2_Cl_2_ (10 mL) and transferred to a separatory funnel. The organic portion was washed with H_2_O (3 × 10 mL), dried (Na_2_SO_4_), filtered and concentrated in vacuo to give a dark yellow oil. The crude material was purified using column chromatography (MeOH: CH_2_Cl_2_ 5:25 v/v) to give the title compound as a yellow oil (74 mg, 43%). *R_f_* = 0.36 (MeOH: CH_2_Cl_2_ 5:25 v/v). ^1^H NMR (CDCl_3,_ 500 MHz): 7.23–7.10 (10H, m), 6.75 (2H, d, *J* = 8.8 Hz), 6.53 (2H, d, *J* = 8.8 Hz), 3.96 (2H, t, *J* = 5.8 Hz), 3.67 (2H, t, *J* = 4.3 Hz), 3.67–3.62 (12H, m), 2.87 (2H, t, *J* = 5.5 Hz), 2.75 (2H, t, *J* = 5.5 Hz), 2.49 (2H, q, *J* = 7.6 Hz), 2.37 (3H, s), 0.91 (3H, t, *J* = 7.6 Hz). HRMS (ESI-TOF) *m*/*z*: [M + H] calcd for C_33_H_44_NO_5_ 534.3219, found 534.3214. Data (Figure S3 in Supplementary Materials) is in accordance with literature [47,48].

### 3.7. Synthesis of (Z)-1-(4-(1,2-Diphenylbut-1-en-1-yl)Phenoxy)-3-Methyl-6,9,12-Trioxa-3-Azatetradecan-14-yl Methanesulfonate 4 

The procedure was adapted from a previous study on the mesylation of alcohols [55]. MeSO_2_Cl (23 µL, 0.287 mmol) was added dropwise into a solution of compound **3** (102 mg, 0.191 mmol), Et_3_N (80 µL, 0.578 mmol) and anhydrous CH_2_Cl_2_ (5 mL) at 0 °C. The solution was stirred under an N_2_ atmosphere for 2 h. The reaction mixture was transferred to a separatory funnel and washed with H_2_O (3 × 10 mL). The organic portion was dried (Na_2_SO_4_), filtered and concentrated in vacuo to give a yellow oil. The crude material was purified using column chromatography (1–10% MeOH in CH_2_Cl_2_) to give the desired product as a colourless oil (45 mg, 13%). *R_f_* = 0.57 (10% MeOH in CH_2_Cl_2_). ^1^H NMR (CD_3_OD, 500 MHz; Figure S4 in Supplementary Materials) δ 7.21–7.11 (10H, m), 6.77 (2H, d, *J* = 8.9 Hz), 6.58 (2H, d, *J* = 8.9 Hz), 4.32 (2H, t, *J* = 4.4 Hz), 3.99 (2H, t, *J* = 5.6 Hz), 3.71 (2H, t, *J* = 4.4 Hz), 3.61–3.71(10H, m), 3.07 (3H, s), 2.86 (2H, t, *J* = 5.9 Hz), 2.73 (2H, t, *J* = 5.9 Hz), 2.45 (2H, q, *J* = 7.5 Hz), 2.38 (3H, s), 0.90 (3H, t, *J* = 7.4 Hz). ^13^C{1H} NMR (CD_3_OD, 125 MHz; Figure S5 in Supplementary Materials) δ 158.2, 145.1, 143.8, 142.7, 139.9, 137.0, 133.0, 130.9, 130.4, 129.2, 129.0, 127.7, 127.2, 114.5, 71.6, 71.54, 71.51, 71.4, 70.9, 69.6, 66.4, 57.8, 57.3, 43.6, 37.5, 29.9, 13.8. HRMS (ESI-TOF) *m*/*z*: [M + H] calcd for C_34_H_46_NO_7_S 612.2950, found 612.2948. Resulting compound achieved > 97% purity (Figure S6 and Table S1 in Supplementary Materials).

### 3.8. Synthesis of MSN-PLH-TAM

PLH-grafted MSNs (33 mg) were ultrasonically dispersed in anhydrous CH_3_CN (1 mL) before a solution of TAM-TEG-OMs 4 (33 mg, 0.054 mmol) in CH_3_CN (3 mL) was added. The suspension was stirred at 80 °C for 24 h. The resulting white precipitate was collected using centrifugation (8000 RPM, 15 min) and washed extensively with CH_3_CN (20 Ml × 3), H_2_O (20 mL × 3), and left to dry under vacuum in a desiccator overnight to give 20 mg of white solid. The combined graft ratio of total organic functionalities (including TAM and PLH) onto MSN surface was determined by TGA (Discovery TGA 550). Approximately 6 mg samples were heated at a rate of 10 °C/min under a flow of nitrogen, from room temperature to 800 °C. The grafting ratio of surface organic functionalities was determined using the equation below:

$$\frac{L1}{100\%\;–\;L1}=\frac{L2\;–\;W}{100\%\;–\;L2\;}$$

where *L*_1_ is the weight loss of native MSNs upon heating to 800 °C, *L*_2_ is the weight loss of fully functionalized MSNs and *W* is the graft ratio [36,56].

## 4. Conclusions

We have successfully synthesized a novel and versatile MSN-based nanostructure, functionalized with the FDA-approved hormonal drug tamoxifen that acts as a guiding vector. The challenging, multistep procedure to synthesize this system required thorough optimization to achieve appropriate grafting densities of both the PLH and TAM. Each step of the synthesis has been extensively characterized to demonstrate the successful fabrication of this novel system. In terms of future applications, our nanostructure can serve as a nanocarrier system for incorporated therapeutic and diagnostic molecules for cancer treatment, given the well-establish property of MSN-based materials to host both hydrophobic and hydrophilic cargoes [26]. For example, a new generation of DDSs derived from our study could combine the advantageous structural properties of MSNs and the safety, targeting ability and efficacy of TAM together with the biocompatibility and high biodegradability of the pH-sensitive material PLH, for potentially enhanced BC treatment outcomes. It is expected that the triple-action system that we have developed holds remarkable promise as a drug delivery vector for cancer therapy. Our future work will investigate the release kinetics and breast cancer targeting capacity, following the encapsulation of our nanostructure with therapeutic and imaging agents used in the treatment and diagnosis of breast cancer.

## Figures and Tables

**Table 1:** Survey XPS data of native MSNs vs. MSN-PLH-TAM.

Sample	SE	At%
Native MSNs (unfunctionalized)	O	66.12
	C	8.44
	Si	25.44
MSN-PLH-TAM	O	38.83
	C	44.77
	N	2.20
	Si	14.20

SE = Surface element. At% = Atomic percentage.

## Supplementary Materials

Figures S1–S4: ^1^H NMR spectra of **1**–**4**; Figure S5: ^13^C NMR spectrum of compound **4**; Figure S6: RP-HPLC chromatogram for **4**; Table S1: RP-HPLC peaks and integrations for **4**.

## Sample Availability

Approximately 50 mg of the native MSNs (Scheme 1a) and MSN-PLH (Scheme 1b) are available from the authors. Compounds 1-4 (Scheme 1c) and MSN-PLH-TAM (Scheme 1d) are only synthesized on demand.
