# Supplementary material for: Concept Design, Development and Preliminary Physical and Chemical Characterization of Tamoxifen-Guided-Mesoporous Silica Nanoparticles
Source: Molecules. 2021 Jan 4;26(1):219. doi: 10.3390/molecules26010219 (PMC7795496; doi:10.3390/molecules26010219)
Supplement: Supplementary file 1 [file molecules-26-00219-s001.pdf]

molecules

MDPI

Supplementary material

Concept design, development and preliminary physical and chemical characterization of Tamoxifen-guided-mesoporous silica nanoparticles

Candace M. Day [1], Martin J. Sweetman [1,2], Shane M. Hickey [1], Yunmei Song [1], Yongjun Liu [3], Na Zhang [3], Sally E. Plush [1,2]* and Sanjay Garg [1,2]*

[1] University of South Australia: Clinical and Health Sciences, North Terrace, Adelaide SA 5000; Candace.Day@mymail.unisa.edu.au, Martin.Sweetman@unisa.edu.edu.au, Shane.Hickey@unisa.edu.au, May.Song@unisa.edu.au, Sally.Plush@unisa.edu.au, Sanjay.Garg@unisa.edu.au.

[2] Future Industry Institute, University of South Australia, Mawson Lakes SA 5095.

[3] School of Pharmaceutical Science, Shandong University, Ji'nan, China; Liuyongjun@sdu.edu.cn, Zhangnancy9@sdu.edu.cn.

* Correspondence: Sally.Plush@unisa.edu.au; Tel.: +61-8-8302-2586; Sanjay.Garg@unisa.edu.au; Tel.: +61-8-8302-1575

Table of Contents

Figures

Figure S1: ^{1}H NMR (500 MHz) spectrum of NMDT **1** in CD_3OD 1

Figure S2: ^{1}H NMR (500 MHz) spectrum **2** in $CDCl_3$ 1

Figure S3: ^{1}H NMR (500 MHz) spectrum of **3** in $CDCl_3$ 2

Figure S4: ^{1}H NMR (500 MHz) spectrum of **4** in CD_3OD 2

Figure S5: ^{13}C NMR (500 MHz) spectrum of **4** in CD_3OD 3

Figure S6. RP-HPLC chromatogram of TAM-TEG-OMs **4** 3

Table S1: RP-HPLC peak table with integrations for TAM-TEG-OMs **4**. 4

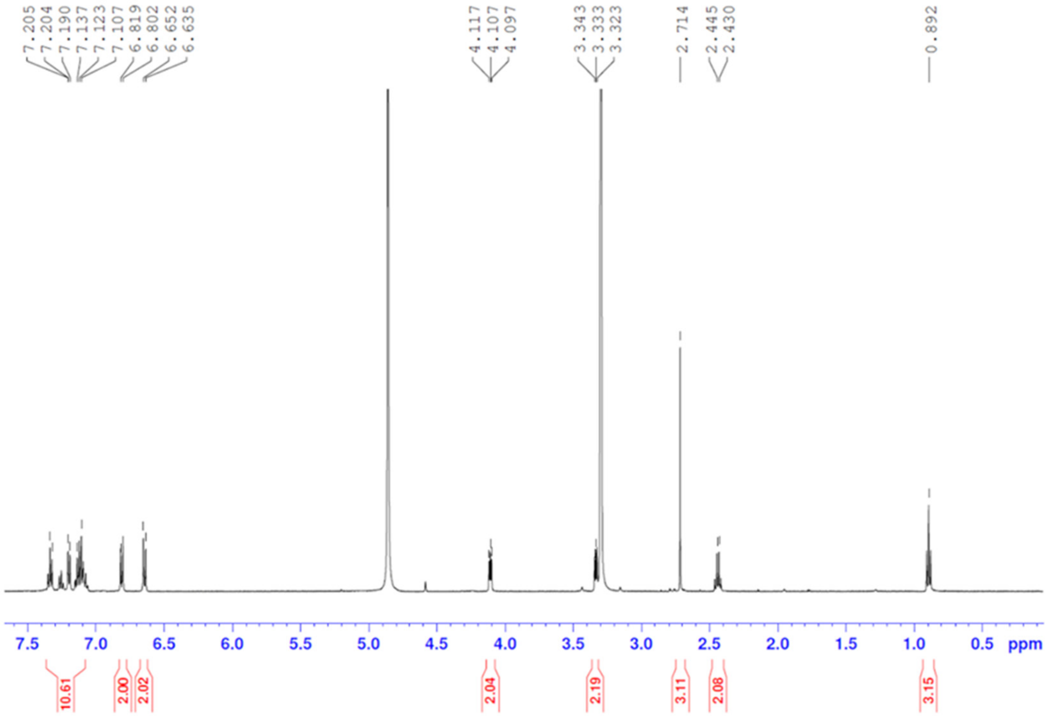

Figure 1. ^{1}H-NMR (500 MHz) spectrum of 1 in CD_3OD.

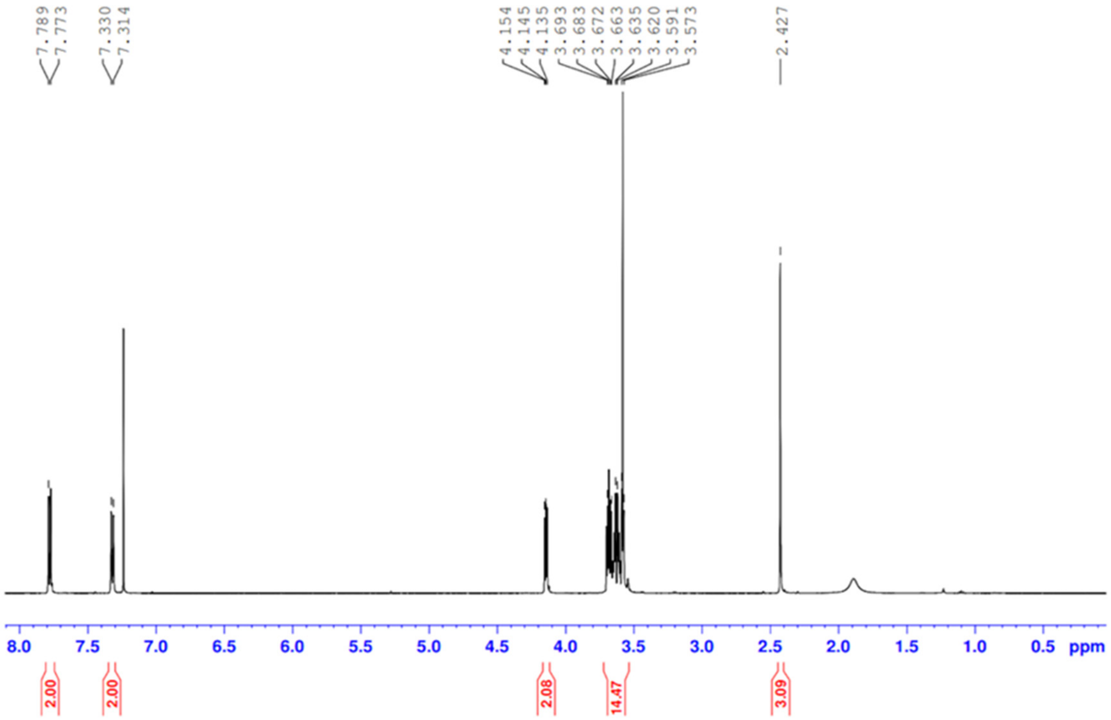

Figure 2. ^{1}H-NMR (500 MHz) spectrum of **2** in $CDCl_3$.

molecules
MDPI

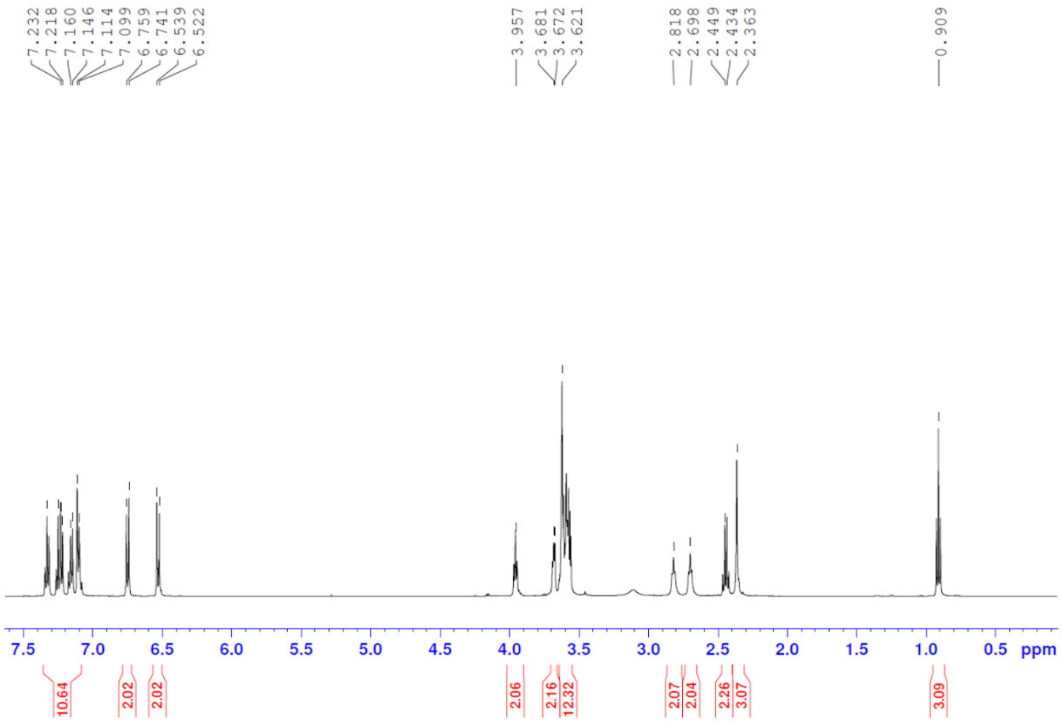

Figure 3. ^{1}H-NMR (500 MHz) spectrum of 3 in $CDCl_3$.

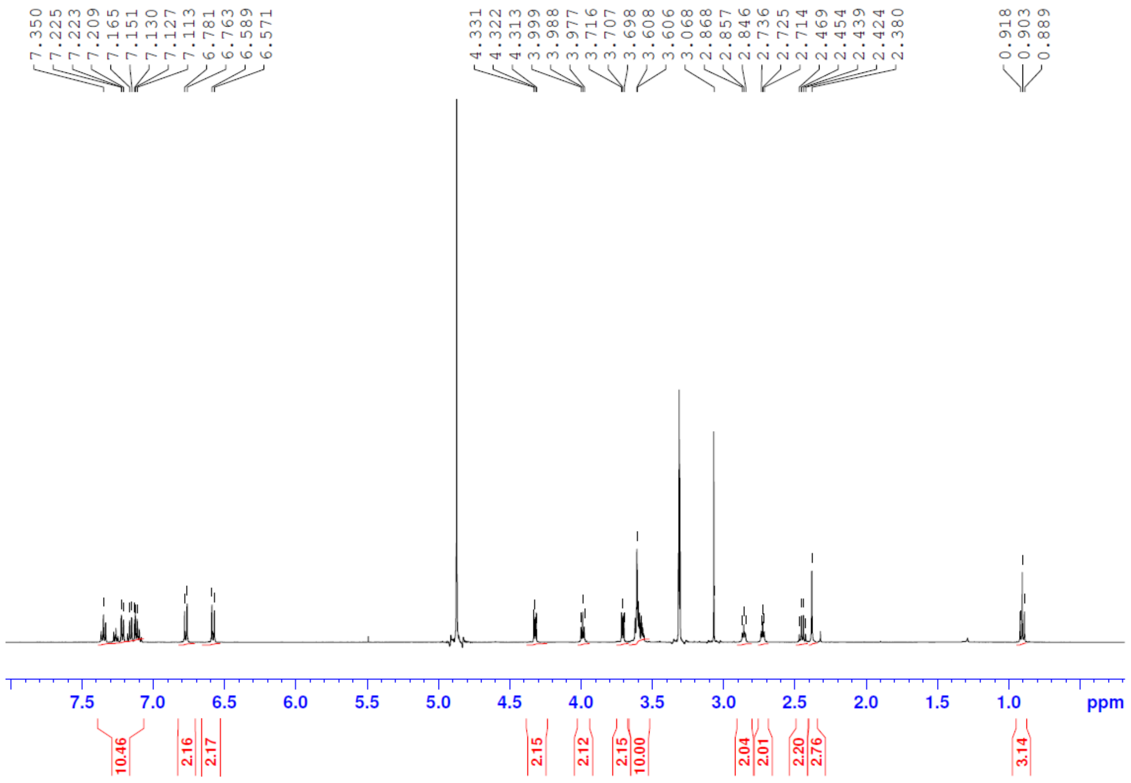

Figure 4. ^{1}H-NMR (500 MHz) spectrum of **4** in CD_3OD.

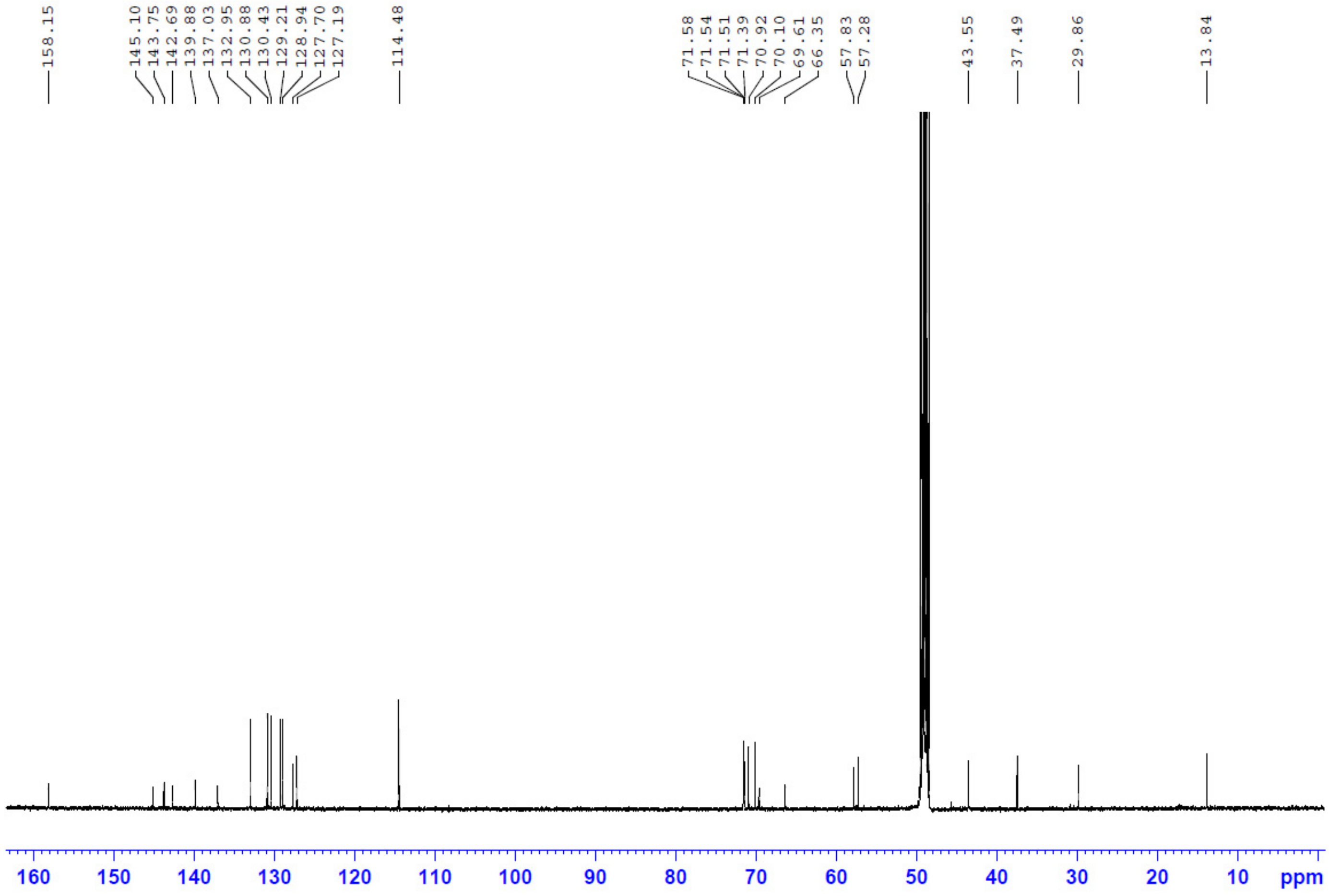

Figure 5. ^{13}C-NMR (125 MHz) spectrum of **4** in CD_3OD.

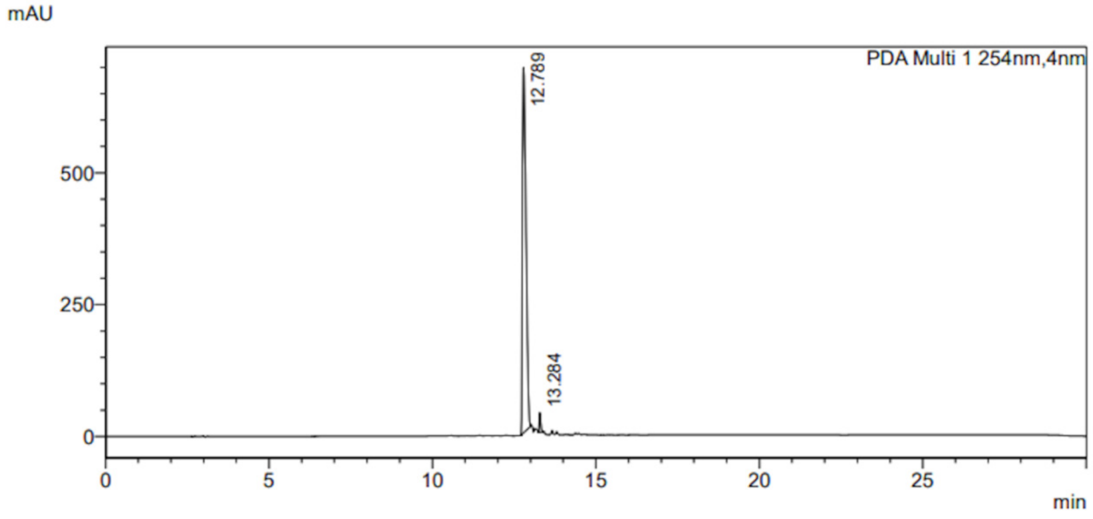

Figure 5. Analytical RP-HPLC chromatogram of TAM-TEG-OMs 4: Chromatography was performed on a Shimadzu HPLC system (Japan), using a C18 column (4.6 × 150 mm, 5 μm) as the stationary phase. The gradient mobile phase was 5–95% MeOH in H_2O. The total run time was 30 min, with flow rate set to 1 mL/min. The injection volume was 50 μL, and the oven temperature was set to 25 °C. Integrations of signals are provided in Table S1.

molecules
MDPI

Table 1. Integrations of RP-HPLC signals for compound **4**.

Peak #	Retention time	Area	Height	Concentration
**1**	12.789	5217958	692731	97.601
**2**	13.284	128267	36842	2.399
**Total**		52346225	729574	
